# Chronic GVHD: predictive factor for rhinosinusitis in bone marrow transplantation

**DOI:** 10.1016/S1808-8694(15)30964-2

**Published:** 2015-10-19

**Authors:** Erica Ortiz, Eulalia Sakano, Carmino Antonio De Souza, Afonso Vigorito, Katia A.B. Eid

**Affiliations:** a Otorhinolaryngologist; b Otorhinolaryngologist, Coordinator of the Rhinology Department – Discipline of Otorhinolaryngology - UNICAMP; c MD. Hematologist. Associate Professor of Hematology - UNICAMP, Head of the Bone Marrow Transplant Department - UNICAMP; d MD. Hematologist. PhD, Professor from the Department of Hematology; e MD. Hematologist. Assistant Physician at the Bone Marrow Transplant Department - UNICAMP. Disciplina de Otorrinolaringologia da UNICAMP. Thanks to Dr. Anibal Faundes, who much contributed to this paper

**Keywords:** GVHD, rhinosinusitis, BMT

## Abstract

**Introduction:**

Bone marrow transplantation (BMT) is a treatment option for hematological diseases and immunodeficiency. It is frequently used today. BMT predisposes patients to upper airway infections and its complications, such as rhinosinusitis (RS). Chemotherapy, radiotherapy, viral infections, antibiotic therapy, graft versus host disease (GVHD) are rhinosinusitis predisposing conditions.

**Aim:**

to investigate RS frequence in this population and its relationship to GVHD; to try and establish the best treatment for RS in these patients.

**Method:**

ENT evaluation of two groups. One group with 35 patients (gI) and another with 24 patients (gII), before and after BMT. They were treated with antibiotics, maxillary sinus punction or endoscopic sinusectomy.

**Results:**

none of them had RS before BMT. 42.8% from gI had RS and 34% had GVHD; in the gII, 58% had RS and 25% had GVHD. 49% from both groups had RS and 30.5% had GVHD. There was significantly more RS in chronic GVHD patients. Surgery was used to treat RS in chronic GVHD patients who underwent BMT.

**Conclusion:**

RS frequence was 49%; GVHD is a predisposing condition to RS; sinusectomy may be necessary to control RS in GVHD patients.

## INTRODUCTION

Bone marrow transplant (BMT) is used as a treatment for blood malignant and non-malignant diseases, immunedeficiencies and solid tumors^1^. Despite enhancements in the techniques and drugs used in current transplants, the bone marrow transplanted patient is prone to multiple upper airway infections and their complications during the first two years after the transplant^2^, ^3^. Immunesupression, radiotherapy, chemotherapy, antibiotic therapy, graft versus host disease (GVHD) and viral infections are described as predisposing factors for these infections and complications^2^, ^3^, ^4^, ^5^, although the exact predictive value is yet to be defined.

We know that acute and chronic rhinosinusitis are frequent in transplanted and immunesupressed patients, specially in allogenic BMT, which donors may be relatives or not1. Although with low incidence (1.7%), invasive fungus rhinosinusitis is the most feared upper airway disease, because its outcome is usually fatal^5^, ^6^, ^7^. Notwithstanding, bacterial rhinosinusitis occur much more frequently and recur more often in these transplanted patients, making its treatment a real challenge. Delays in diagnosis and treatment of these rhinosinusitis may cause a fatal complication or BMT failure.

Treatment for recurrent or chronic bacterial rhinosinusitis in BMT patients differs from that in immunecompetent patients, because a number of factors contribute to maintain nasosinusal mucosal alterations and immunity. Among these factors, as the ones mentioned above, chronic Graft Versus Host Disease (cGVHD) seems to play an important role. It is known that chronic GVHD occurs at about 3 months following BMT in approximately 30% of the patients, while their immunity reacts favorably. There is gastrointestinal and respiratory mucosal involvement, among other organs, through a T lymphocyte reaction against class II antigens of these mucosal cells, causing local inflammation. Chronic GVHD may be classified into limited or extensive forms, depending on the number of organs involved. The better the patient's immunity, the greater is the mucosal inflammatory reaction. Therefore, patients who develop chronic GVHD are medicated with immunesupressants to control this reaction, and this maintains a borderline immunity.

According to the North American and Latin American Rhinosinusitis consensus, acute rhinosinusitis should be treated with antibiotics; and the chronic with anti-inflammatory, antihistamine, decongestant medications, or even endonasal surgeries depending on the disease maintenance factor. For BMT the treatment advocated is based on broad spectrum antibiotics due to Immunesupression. However, one must consider local factors such as severe mucosal edema due to the inflammation caused by chronic GVHD. In these situations it is likely that the surgical approach would be beneficial.

This paper aims at studying the frequence of rhinosinusitis after bone marrow transplant and to study the association between rhinosinusitis and chronic graft versus host disease, and also the need for surgery to treat rhinosinusitis in BMT patients.

## MATERIALS AND METHODS

This is a retrospective study, carried out between September of 2001 and June of 2003, at the Otolaryngology and Hematology Departments of the University Hospital – State University of Campinas. The Ethics Committee for Research of the School of Medical Sciences of the Medical School UNICAMP approved this research project and we obtained an informed consent from all the participant patients.

59 patients with hematological diseases and BMT indication were assessed. They were divided in two groups. Group I comprised 35 patients who were assessed and followed before and after BMT. Group II, with 24 patients, was assessed only after BMT.

The assessment followed a pre-established protocol of otorhinolaryngological interview, physical exam (otoscopy, rhinoscopy, oroscopy, neck palpation), nasosinusal CT scan (coronal cross section) and/or paranasal sinus X-rays. Paranasal sinus CT scan was carried out in 30 patients from group I and only in 6 patients from group II.

Rhinosinusitis diagnosis was based on the parameters described by the North American and Latin American Consensus on Rhinosinusitis, in other words, we investigated time of evolution, symptoms (fever, facial pain, cough, nasal congestion and purulent rhinorrhea), purulent secretion in the meatus seen at nasal endoscopy and radiological alterations (paranasal sinus veiling). Paranasal sinuses CT scan was carried out in 21 of all the patients who were diagnosed with rhinosinusitis and paranasal sinuses X-ray was done in 8 patients.

Treatment was based on antibiotics as defined by the Hospital Infections Control Committee – University Hospital UNICAMP. When the patient presented with refractory, recurrent or chronic rhinosinusitis with intranasal and/or paranasal sinus anatomical alterations (maxillary cyst, septal deviation, bullous conchae, polyposis, tumor) this patient underwent maxillary sinus punction through the canine fossa under local anesthesia for sinusal rigid endoscopy, mucosal and maxillary sinus biopsy and secretion harvesting with sterile catheter introduced through the punction ostium for bacterial culture purposes; or, surgical removal of the obstructive factor in the middle meatus (maxillary, ethmoidal or sphenoidal endoscopic sinusectomy), under general anesthesia. Patients were with high risk of intense nasal bleeding, because of acute myeloid leukemia and total bone marrow aplasia. Patients who did not present nasosinusal disease were only followed. In Group I, the assessment was repeated after BMT and the treatment was carried out according to the alterations presented at the moment.

Data statistical analysis was carried out through averages, medians, Yales-corrected chi-squared and Fisher Test.

## RESULTS

Group I, which was assessed before and after BMT, comprised 35 adult patients with average age of 35 years. Bone marrow transplant for this group was mostly allogenic (88%). None of them had rhinosinusitis before BMT. After BMT, 15 patients (42.8%) had rhinosinusitis. In this group, 12 (34%) patients developed chronic GVHD. There was no statistical significance between GVHD and rhinosinusitis. Notwithstanding, we noticed that over half of the patients with chronic GVHD had rhinosinusitis, while only a quarter of the patients without chronic GVHD presented with rhinosinusitis (Table 1).

**Table 1:** Relationship between rhinosinusitis (RS) and chronic graft versus host disease (cGVHD) for group I.

	WITH cGVHD	WITHOUT cGVHD			P
With RS	7(58%)	8(34%)	Chi-squared	1,785929952	0,181422943
Without RS	5(41%)	15(65%)	Corrected	0,953728865	0,32877197
Total	12	23	Fisher test	0,164479051	

Group II, assessed after BMT, comprised of 24 patients of the same age range as those in group I, the type of transplant most frequently done was also the allogenic. In this group, 14 patients (58%) had rhinosinusitis; and six (25%) had cGVHD. We saw that all the patients with GVHD developed rhinosinusitis, and there was statistical significance (Table 2).

**Table 2:** Relationship between rhinosinusitis (RS) and chronic graft versus host disease (cGVHD) for group II.

	WITH cGVHD	WITHOUT cGVHD			P
With RS	6(100%)	8(44%)	Chi-squared	5,714285714	0,016827411
Without RS	0	10(55%)	Corrected	3,657142857	0,055829302
total	6	18	Fisher test	0,022311213	

Since both groups presented similar characteristics as to the frequence of GVHD, we repeated the analysis in the set of both groups. (Table 3). The frequence of rhinosinusitis, in the total of 59 patients, was of 49% (29 patients). GVHD occurred in 18 patients (30.5%). Rhinosinusitis in the patients with GVHD is more frequent. Over two-thirds of the patients with chronic GVHD had rhinosinusitis; on the other hand, less than half of the patients without cGVHD had rhinosinusitis. There was statistical significance between rhinosinusitis and cGVHD.

**Table 3:** Relationship between rhinosinusitis (RS) and chronic graft versus host disease (cGVHD) in all patients.

	WITH cGVHD	WITHOUT cGVHD			P
With RS	13	16	Chi-squared	5,515800704	0,018845446
Without RS	5	25	Corrected	4,267474613	0,03884863
Total	18	41	Fisher test	0,018752318	

Antibiotics were used for treatment in all the cases; however, some patients underwent maxillary, ethmoid and/or sphenoid sinusectomy to better control their rhinosinusitis. We can see that a little bit more than half of the patients with cGVHD who had rhinosinusitis needed endoscopic sinusectomy to control the disease, while only one third of the patients without cGVHD underwent such surgery. However, there was no statistical significance in relation to surgery indication and rhinosinusitis among the cGVHD with BMT patients (Table 4).

**Table 4:** Relationship between drug therapy (antibiotics) and surgery (FESS) and cGVHD.

	WITH cGVHD	WITHOUT cGVHD		P	
Antibiotics	6 (46%)	11 (69%)	Chi-squared	1,50973322	0,219179923
surgery	7 (54%)	5 (31%)	Corrected	2,584965356	0,107882796
Total	13	16	Fisher test	0,197969436	

## DISCUSSION

Rhinosinusitis in transplanted patients is inevitable due to multiple intrinsic predisposing factors of the transplant process (whole body radiotherapy, conditioning, long term antibiotic treatment, neutropenia, graft versus host disease, chemotherapy). Besides the major alteration, which is immunedeficiency in these patients, Cordonnier et al. showed cilliary alterations in the nasal mucosa that may predispose these patients to bacterial recurrent rhinosinusitis. The likelihood of the patient developing rhinosinusitis in the first two years after BMT is estimated to be up to 36.9%, according to Savage et al., going against findings of 49% of frequence in this paper.

According to numerous authors^8^, ^9^, ^10^, ^11^, ^12^, otorhinolaryngological physical exam alone is not enough to properly assess these transplanted patients. Nasal endoscopy and paranasal sinus CT scan before BMT are ideal and fundamental tests used for the precise and early diagnosis. This way it is possible to know precisely of anatomical nasal conditions, nasal mucosa situation and paranasal sinus ostia, and to find tumors in the posterior nasal region. After BMT, endoscopy is the best test for a precise follow up of these transplanted patients, because there is a need to differentiate secretion color, mucosal edema or early fungi manifestations (gray, yellow or dark mucosa) in the entire nasal cavity. In suspicious cases of bacterial or fungi rhinosinusitis after BMT, it is possible to repeat the CT scan in order to plan treatment and disease staging.

The occurrence of chronic graft versus host disease (cGVHD) is estimated to be of 60% in the patients submitted to allogenic transplants^1^, ^2^, ^4^. In this paper, the frequence found was low (30.5%). We know that the cGVHD patients may have limited or extensive lesions (liver, skin, lungs, eyes) through auto-reactive T lymphocytes from the donor against class II antigen molecules common to the receiver^1^. In the lungs, obliterating bronchiolitis is the typical manifestation^2^. We have no specific report about nasosinusal mucosa alterations caused by cGVHD, but rather of the oral cavity mucosa, where we find important edema and lymphocyte build up in the submucosa. There is also association of immunedeficiency both humoral and cellular, and the use of immunosuppressive drugs to avoid the progression of cGVHD itself^2^. Cordonnier was not able to relate nasal mucosa cilliary alterations to cGVHD development.

It is very likely that the airways are the first infection site because of its easy contact and contamination through the environment, having seen that immunity is deficient. Notwithstanding, the other factors, including Graft Versus Host Disease, participate in the maintenance of inflammation in the upper airways and rhinosinusitis recurrence. Thus, this paper has shown the influence of cGVHD in rhinosinusitis. It may be that the cilliary alterations caused by cGVHD would cause the need for surgery in order to broaden the facial sinuses ostia, thus enhancing aeration and reducing secretion retention within the paranasal sinuses. Although this paper has failed to show any significance between paranasal sinus surgery indication and rhinosinusitis in the cGVHD BMT patients, we have seen clinically that these patients did not have more rhinosinusitis recurrences in the 2 year follow up period. Therefore, at the University Hospital -UNICAMP we chose to do nasosinusal endoscopic surgery in cGVHD BMT patients who had recurrence of rhinosinusitis (> 2-3 episodes in the semester) or chronic rhinosinusitis, or therapy failure even if it happened in the first episode.

## CONCLUSION

Rhinosinusitis frequence in BMT patients was of 49%.

The frequence of chronic Graft Versus Host Disease was of 30.5%.

Chronic Graft Versus Host Disease is a predisposing factor for rhinosinusitis.

Nasosinusal surgery may be necessary to control rhinosinusitis in patients with chronic Graft Versus Host Disease.
